# Air pollution enhance the progression of restrictive lung function impairment and diffusion capacity reduction: an elderly cohort study

**DOI:** 10.1186/s12931-022-02107-5

**Published:** 2022-07-14

**Authors:** Chi-Hsien Chen, Chih-Da Wu, Ya Ling Lee, Kang-Yun Lee, Wen-Yi Lin, Jih-I Yeh, Hsing-Chun Chen, Yue-Liang Leon Guo

**Affiliations:** 1 Department of Environmental and Occupational Medicine, National Taiwan University Hospital Hsin-Chu Branch, No. 25, Ln. 442, Sec. 1, Jingguo Rd., North Dist., Hsinchu City, Taiwan; 2 Department of Environmental and Occupational Medicine, National Taiwan University (NTU) College of Medicine and National Taiwan University Hospital, Rm 339, No. 7, Zhongshan S. Rd., 17 Syujhou Road, Zhongzheng Dist., Taipei City, 100 Taiwan; 3 Department of Geomatics, National Cheng Kung University, No. 1, Daxue Rd., East Dist., Tainan City, Taiwan; 4 National Institute of Environmental Sciences, National Health Research Institutes, No. 35, Keyan Rd., Zhunan Township, Miaoli County Taiwan; 5 Department of Dentistry, Taipei City Hospital, No. 33, Sec. 2, Zhonghua Rd., Zhongzheng Dist., Taipei City, Taiwan; 6 Department of Dentistry, School of Dentistry, National Yang Ming Chiao Tung University, No. 155, Sec. 2, Linong St., Beitou Dist., Taipei City, Taiwan; 7 University of Taipei, No. 1, Aiguo W. Rd., Zhongzheng Dist., Taipei City, Taiwan; 8 Division of Pulmonary Medicine, Department of Internal Medicine, Shuang Ho Hospital, Taipei Medical University, No. 291, Zhongzheng Rd., Zhonghe Dist., New Taipei City, Taiwan; 9 Department of Internal Medicine, School of Medicine, College of Medicine, Taipei Medical University, No. 250, Wuxing St., Xinyi Dist., Taipei City, Taiwan; 10 Department of Occupational Medicine, Health Management and Occupational Safety Hygiene Center, Kaohsiung Municipal Siaogang Hospital, No. 482, Shanming Rd., Siaogang Dist., Kaohsiung City, Taiwan; 11 Research Center for Environmental Medicine, Kaohsiung Medical University, No. 100, Shih-Chuan 1st Rd., Sanmin Dist., Kaohsiung City, Taiwan; 12 Department of Family Medicine, Hualien Tzu-Chi General Hospital, No. 707, Sec. 3, Zhongyang Rd., Hualien City, Hualien County Taiwan; 13 Division of Pulmonary Medicine, Department of Internal Medicine, Dalin Tzu Chi Hospital, No. 2, Minsheng Rd., Dalin Township, Chiayi County Taiwan; 14 Institute of Environmental and Occupational Health Sciences, National Taiwan University College of Public Health, No. 17, Xuzhou Rd., Zhongzheng Dist., Taipei City, Taiwan

**Keywords:** Air pollution, Nitrogen dioxide, Particulates, Lung function, Total lung capacity, Residual volume, Diffusion capacity, Elderly

## Abstract

**Background:**

Some evidences have shown the association between air pollution exposure and the development of interstitial lung diseases. However, the effect of air pollution on the progression of restrictive ventilatory impairment and diffusion capacity reduction is unknown. This study aimed to evaluate the effects of long-term exposure to ambient air pollution on the change rates of total lung capacity, residual volume, and diffusion capacity among the elderly.

**Methods:**

From 2016 to 2018, single-breath helium dilution with the diffusion capacity of carbon monoxide was performed once per year on 543 elderly individuals. Monthly concentrations of ambient fine particulate matters (PM_2.5_) and nitric dioxide (NO_2_) at the individual residential address were estimated using a hybrid Kriging/Land-use regression model. Linear mixed models were used to evaluate the association between long-term (12 months) exposure to air pollution and lung function with adjustment for potential covariates, including basic characteristics, indoor air pollution (second-hand smoke, cooking fume, and incense burning), physician diagnosed diseases (asthma and chronic airway diseases), dusty job history, and short-term (lag one month) air pollution exposure.

**Results:**

An interquartile range (5.37 ppb) increase in long-term exposure to NO_2_ was associated with an additional rate of decline in total lung volume (− 1.8% per year, 95% CI: − 2.8 to − 0.9%), residual volume (− 3.3% per year, 95% CI: − 5.0 to − 1.6%), ratio of residual volume to total lung volume (− 1.6% per year, 95% CI: − 2.6 to − 0.5%), and diffusion capacity (− 1.1% per year, 95% CI: − 2.0 to − 0.2%). There is no effect on the transfer factor (ratio of diffusion capacity to alveolar volume). The effect of NO_2_ remained robust after adjustment for PM_2.5_ exposure.

**Conclusions:**

Long-term exposure to ambient NO_2_ is associated with an accelerated decline in static lung volume and diffusion capacity in the elderly. NO_2_ related air pollution may be a risk factor for restrictive lung disorders.

## Background

Lung function level and decline rate are known to be predictors for mortality and morbidity [1–3]. Research evidence has shown the long-term effects of air pollution on longitudinal changes in lung function. In children, exposure to nitric dioxide (NO_2_), fine particulate matter (PM_2.5_), acid vapor, elemental carbon, and traffic reduces the growth rate of lung function [4–7]. Among adults and the elderly, exposure to black carbon and PM_2.5_ increases the rate of decline in lung function [8–10].

Previous epidemiological studies have used spirometry to measure forced lung function parameters, mainly forced expiratory volume in one second (FEV1) and forced vital capacity (FVC), to obtain diagnostic information on conductive airway function and the severity of obstruction. An obstructive disorder is defined as a reduced FEV1/FVC ratio below the 5th percentile of the predicted value [11]. Given the limitations of such measurement, diagnosis of restrictive ventilation defect and functional impairment of restrictive lung disorders will require other test methods, such as plethysmography, inert gas dilution, nitrogen washout, and single-breath carbon monoxide uptake, to measure total lung capacity and diffusion capacity [12]. A restrictive ventilatory defect is characterized by a reduction in total lung capacity (TLC) below the 5th percentile of the predicted value, and a normal FEV1/FVC [11]. Disease entities like interstitial lung diseases (ILD) involve regions below the conductive airway and often lead to restrictive ventilation and impaired gas diffusion [11]. Restrictive ventilatory pattern and reduced diffusion capacity are known predictors for mortality risk in healthy people and those with chronic lung diseases.

In recent years, several studies have shown an association between exposures to ambient air pollution and the development or exacerbation of ILD. Johannson et al. found that short-term (six weeks) exposures to ambient ozone and NO_2_ increased the risk of acute exacerbation of idiopathic pulmonary fibrosis (IPF) [13]. Sack et al. reported the association between 10-year exposure to nitrogen oxide (NOx) and an increased risk for subclinical ILD diagnosed by CT imaging [14]. Conti et al. demonstrated the association between NO_2_ and the increased incidence of IPF identified in healthcare administrative databases [15], while Singh et al. reported an increased proportion of hypersensitivity pneumonitis to cases of ILD related to PM_2.5_ exposure [16].

Although imaging can aid in diagnosing interstitial lung disease, it cannot provide an assessment of physiological function. Current knowledge on the effects of air pollution exposure on total lung capacity and diffusing capacity is limited and mixed. Some animal [17–20] and human [21–34] studies have shown that exposure to air pollution has adverse effects on total lung capacity [17, 23, 24, 26–28] and diffusing capacity [19, 20, 23, 24, 29–33], but others have shown no effects [18, 21, 22, 25, 34]. Most previous studies have focused on acute [20, 25, 28–30, 32–34] or short-term [21–24] effects, but only a few have reported long-term effects [17–19, 26, 27, 31]. No studies have reported the long-term effect of air pollution exposure on the decline total lung capacity and diffusion capacity.

Older people are susceptible to air pollution and the development of pulmonary fibrosis due to the lower anti-oxidative enzyme activity [35] and shorter telomere length [36]. Among the elderly, restrictive ventilator defect, lung function decline, and diffusion capacity are predictors of mortality and morbidity [37–39]. However, no study has reported the long-term effect of air pollution exposure on the longitudinal changes of these lung function indicators among the elderly. Therefore, this study of an elderly cohort aimed to examine the long-term effects of air pollution on static lung volume and diffusion capacity of the lungs.

## Methods

### Subjects

This cohort study followed-up 1496 elderly individuals (age > 65 years old) from 2016 to 2018. They were invited during the annual health check-up for the elderly in five hospitals in Taiwan. To maximize differences in exposure, the hospitals were selected from five areas with varying levels of air pollution, including one from Eastern Taiwan (Hualien Tzu-Chi Hospital), two from Northern Taiwan (Taipei City Hospital and Shuang Ho Hospital), and two from SouthWest Taiwan (Siaogang Hospital and Dalin Tzu Chi Hospital). Of the 1496 participants, 543 were randomly sampled and examined every year for the diffusing capacity of the lungs for carbon monoxide (DLco). The institutional review boards of all participating institutions approved the study protocol and all of the participants provided written informed consent.

### Questionnaire evaluation

A questionnaire was used to collect personal information on physician-diagnosed diseases (e.g., asthma and chronic airway diseases), conditions of regular medical treatment for chronic diseases, smoking status, educational attainment, exposures to indoor air pollution (e.g., second-hand smoke, cooking fume, and incense), and occupational history. The frequency of exposure to indoor air pollution and the duration of each exposure were also assessed. All participants were asked, “How many times have you inhaled secondhand smoke in the past month?”, “How many meals have you cooked with frying, stir-frying, or deep-frying by yourself in the past month?”, and were asked to rate frequency on eight levels, “three or more times a day, twice a day, once a day, 3–6 times a week, 1–2 times a week, 1–3 times a month, less than once a month, or never”, and to report the average duration (in minutes) of each exposure or activity. Secondhand smoke refers to the smoke from burning cigarettes or exhaled by smokers in the same residence. In addition, kitchen ventilation is assessed by asking, “In the past week, did you and your family turn on the range hood when cooking with frying, stir-frying, or deep-frying (excluding boiling)?”. Subjects were also asked, “How many times does your family burn incense to the gods and ancestors in a week?” and rated the frequency on four levels, “more than three times a week, 1–3 times a week, less than once a week, or never”. For assessing occupational history, we asked subjects about their past work experience (more than one year), including occupation, job title, and tenure. We defined wood product manufacturing, manufacturing of leather, fur and its products, metal products manufacturing, agriculture, rubber product manufacturing, construction industry, mining, and on land clay and stone quarrying as occupations exposed to dusty environment dust. A “dusty job history” was defined as working experience in the abovementioned dusty occupations with job titles of semi-professional, skilled, or semi-skilled worker for more than ten years.

A questionnaire evaluation was conducted on each visit throughout the follow-up period. To facilitate the answering of the questionnaire, all of the participants had face-to-face interviews with five well-trained research assistants.

### Measurements of forced lung function

The participants underwent forced pulmonary function testing within one month before measuring the diffusion capacity. Diffusion testing was delayed because it required some administrative time to schedule tests in hospital labs. Five well-trained research assistants used spirometers in field surveys and performed the test following the standard protocol of the American Thoracic Society [40]. Briefly, participants were tested in a seated position using a nose clip and instructed to exhale forcefully immediately after taking a deep breath and continue to exhale until the volume-time curve reached a plateau of 1–2 s. Acceptable blow criteria included (1) good expiratory initiation without excessive hesitation or false initiation; (2) no coughing or glottis closure within the first second of exhalation; (3) no early termination of expiration; (4) no leakage; (5) no mouthpiece obstruction; (6) no additional breathing during the maneuver. This exhalation maneuver was repeated until at least 3 acceptable blows were completed and the difference in volume between the maximum two breaths should be within 5% or < 150 mL. Results with the largest sum of forced vital capacity (FVC) and forced expiratory volume in one second (FEV1) among the repeats were used for data analysis.

### Measurement of static lung function and diffusion capacity

Static lung volume and diffusion capacity of carbon monoxide in the lungs were measured by the single-breath method in the pulmonary function laboratories of the five participating hospitals. During the test, the participants were in a sitting position with a nose clip in place. First, they were asked to do tidal breathing for a sufficient time to ensure that they had adapted to the mouthpiece and oral breathing maneuver. Then, the single-breath maneuver began with unforced exhalation to residual volume, followed by a rapid inhalation of a test gas (21% oxygen, 10% helium, and 0.3% carbon monoxide) to total lung capacity. For the total lung capacity, the participants were required to hold the breath for 10 ± 2 s and then exhale to residual volume.

The parameters in the test reports included total lung capacity (TLC, in liters), residual volume (RV, in liters), diffusion capacity of carbon monoxide (DLco, in ml/min/mmHg), and alveolar volume (VA, in liters). However, one hospital did not provide the first two parameters. The ratio of RV/TLC and the carbon monoxide transfer coefficient (DLco/VA) were also calculated.

### Assessment of air pollution exposure

The monthly levels of PM_2.5_ and NO_2_ at each participant's residential address were estimated using a hybrid spatial model, which incorporated the Kriging interpolated estimates with the land-use regression (LUR) model. The models were constructed based on the daily air pollutant data from the 73 EPA monitoring stations in Taiwan. Predictors for the PM_2.5_ LUR model included kriging-based PM_2.5_ estimates, concentrations of NO_2_, SO_2_, and O_3_, area of fruit orchard within a 1750 m buffer radius, area of paddy field and fruit orchard within a 2000 m buffer radius, the industrial area within 750 m buffer radius, number of temples within a 500 m buffer radius, relative humidity, and the minimum value of the Normalized Difference Vegetation Index (NDVI) within a 1500 m buffer radius. On the other hand, predictors for the NO_2_ LUR model included kriging-based NO_2_ estimates, concentrations of PM_10_, SO_2_, and O_3_, the total length of major roads within 25 m and 5000 m buffer radii, the total length of local road within a 750 m buffer radius, area of water within a 5000 m buffer radius, area of farmland and orchard within a 250 m buffer radium, purely residential area within a 4000 m buffer radius, industrial residential area within a 250 m buffer radius, the industrial area within a 5000 m buffer radius, number of temples within a 100 m buffer radius, funeral facility within 100 m and 4000 m buffer radii, nearest distance to the thermal power plant, the mean value of the NDVI within a 1000 m buffer radius, and the maximum value of the NDVI within a 5000 m buffer radius. Monthly PM_2.5_ and NO_2_ concentrations were estimated based on the abovementioned models and mapped at 250 × 250 m grid resolution using ArcGIS software. Personal exposure levels were extracted at their residential address from the maps. The performance of PM_2.5_ and NO_2_ models was evaluated using tenfold cross-validation with explanatory power R^2^ of 0.88 and 0.87, respectively [41, 42]. Long-term air pollution exposure was calculated by averaging the concentrations of each pollutant in the 12 months preceding each lung function measurement.

### Statistical analysis

A linear mixed model with a subject-specific random intercept was used to evaluate the relationship between air pollution and lung function. In the model, an interaction term between the air pollution exposure level and the follow-up duration (in years) of each visit was added. The estimated coefficient referred to the additional annual decline rate of lung function indices related to the exposures, whereas the coefficient of air pollution exposure referred to its cross-sectional relationship with lung function. Lung function indices were logarithmically transformed to normalize the data for analysis. The estimated coefficient was reported as the percentage difference in lung function for an interquartile range (IQR) increment in exposure levels of air pollution. The percentage difference was calculated using the following formula:

$$\left( {{\text{e}}^{\beta } {-}{ 1}} \right) \, \times { 1}00\%$$

where e^β^ was the exponential function of corresponding coefficients.

All models were adjusted for a core set of fixed and time-varying covariates. Fixed covariates include sex, age at baseline, educational attainment, and dusty job history. Time-varying covariates include body height, body weight, follow-up duration, current smoking status, past smoking history, pack-years of smoking, physician-diagnosed diseases (current asthma and chronic airway diseases), obstructive ventilation, second-hand smoke exposure, cooking (frying or stir-frying), use of range hood when cooking (frying, stir-frying, or deep-frying), incense burning, and short-term exposure (lag one month) to air pollution. Missing data of time-varying covariates accounted for 1.1% of the dataset and were replaced with the last visited value. Obstructive ventilation was defined as a pre-bronchodilator FEV1/FVC ratio less than the lower limit of normal value calculated using the Global Lung Initiative 2012 equation, with adjustments for Southeast Asians [43]. The exposure conditions of second-hand smoke and cooking were presented as minutes in a month and were calculated by multiplying the frequency of exposure and the average time (in min) of each exposure. We converted the eight frequency levels to approximately monthly times (i.e., 90, 60, 30, 19, 6, 2, 1, or 0 times a month) before calculating.

All statistical analyses were performed using the JMP pro 13.0.0 (SAS Institute Inc., USA). Statistical significance was set at a two-tailed *P* value of < 0.05.

## Results

**Fig. 1:**
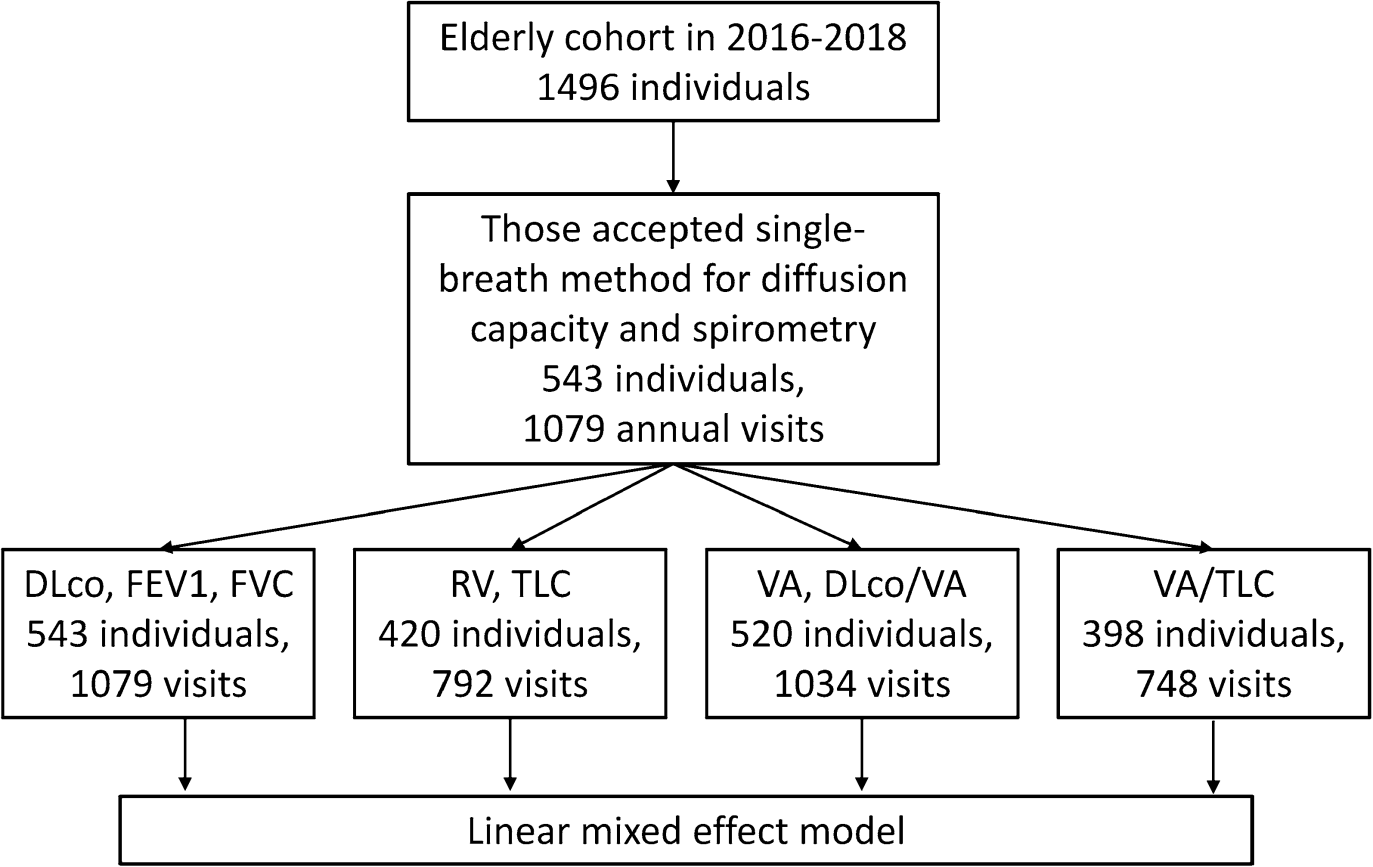
Flowchart of the study design

Figure 1 shows the flowchart of the study design, including case numbers of different lung function indices. Table 1 compares the basic characteristics of subjects who participated and those who did not participate in diffusion capacity testing. The general characteristics of participants and non-participants were the similar, except for age (69.5 versus 70.4 years), education (< 13 years of education, 48% versus 59%), and dusty job history (16% versus 20%). Table 2 presents the basic characteristics of the subjects who participated in the diffusion capacity testing, stratified by age (≤ 70 or > 70 years) and sex. Of the 543 participants who were examined for diffusion capacity of carbon monoxide from 2016 to 2018, 350 underwent more than one visit (mean follow-up duration: 1.8 years; range: 0.3–2.7 years). Of the 420 participants with information on TLC and RV, 165 (39%) had one visit, 138 (33%) had two visits, and 117 (28%) had three visits. From the characteristics of participants on their first visit (Table 1), the mean baseline age was 69.5 years. There were more females (60%) than males (40%). Only 12% had a college or higher level of education and 48% (55% in females and 37% in males) did not reach secondary education. Women spend three times as long cooking with frying, stir-frying, or deep-frying as men (924 min versus 277 min per month). The majority never smoked (82%) and only 5% were active smokers at the time of their first visit. The rate of ever smoking habits in males is much higher than in females (42% versus 2%). Approximately 16% reported dusty job history. Male and older participants have a higher rate of being involved in dusty jobs. Approximately 6% had obstructive ventilation and higher in males than females (8% versus 4%).

**Table 1:** Characteristics of subjects participated and not participated in this study

	Participants	Non-participants	P-value
	n = 543	n = 953	
Baseline characteristics			
Age, mean ± SD, year	69.5 ± 4.1	70.4 ± 4.28	< 0.0001
Male, n (%)	216 (39.8)	417 (43.8)	0.134
Height, mean ± SD, cm	158.1 ± 7.9	157.8 ± 7.9	0.470
Weight, mean ± SD, kg	61.9 ± 10.0	61.4 ± 10.0	0.339
Education < 13 year, n (%)	262 (48.3)	566 (59.4)	< 0.0001
Smoking status, n (%)			
Never	445 (82.0)	783 (82.2)	0.855
Former	72 (13.3)	130 (13.64)	
Current	26 (4.8)	40 (4.2)	
Pack-years*, mean ± SD	18.5 (13.3)	20.3 (15.7)	0.325
Dusty job history, n (%)	86 (15.8)	190 (19.9)	0.049
SHS exposure, min per month, mean ± SD	160.6 ± 1154.4	245.6 ± 1780.2	0.318
Cooking (frying), min per month, mean ± SD	666.5 ± 1175.2	721.8 ± 1389.0	0.435
Incense burning at home, n (%)			
Never	258 (47.5)	433 (45.4)	0.382
< Once a week	10 (1.8)	29 (3.0)	
Once to trice a week	60 (11.1)	121 (12.7)	
> Trice a week	215 (39.6)	370 (38.8)	
Using range hood when cooking (frying), n (%)	482 (88.8)	846 (88.8)	0.997
Asthma, n (%)	13 (2.4)	20 (2.1)	0.708
Regular treatment for asthma, n (%)	6 (1.1)	12 (1.3)	0.795
Chronic airway diseases, n (%)	14 (2.6)	17 (1.8)	0.300
Regular treatment for chronic airway diseases, n (%)	7 (1.3)	12 (1.3)	0.960

*SHS* second-hand smoke, *SD* standard deviation, *COPD* chronic obstructive pulmonary disease

*Pack-years was calculated only for smoker and ex-smoker

**Table 2:** Characteristics of the 543 elderly participating in 1079 visits in this study, 2016–2018

	Female		Male	
	≤ 70 years	> 70 years	≤ 70 years	> 70 years
	n = 225	n = 102	n = 109	n = 107
Baseline characteristics				
Age, mean ± SD, year	66.8 ± 1.7	73.6 ± 2.6	66.7 ± 1.6	74.2 ± 2.9
Height, mean ± SD, cm	154.3 ± 5.6	152.5 ± 5.2	165.5 ± 6.2	164 ± 6
Weight, mean ± SD, kg	58.6 ± 8.7	56.9 ± 7.2	69.5 ± 9.5	66 ± 9.5
Education < 13 year, n (%)	113 (50.2)	69 (67.6)	44 (40.4)	36 (33.6)
Smoking status, n (%)				
Never	219 (97.3)	101 (99)	67 (61.5)	58 (54.2)
Former	5 (2.2)	1 (1)	32 (29.4)	34 (31.8)
Current	1 (0.4)	0 (0)	10 (9.2)	15 (14)
Pack-years*, mean ± SD	18.4 ± 5.1	17.6	19.2 ± 16.1	17.9 ± 11.6
Dusty job history, n (%)	24 (10.7)	14 (13.7)	22 (20.2)	26 (24.3)
SHS exposure, min per month, mean ± SD	159.6 ± 1447.7	155.4 ± 651.2	202.4 ± 1154.3	125 ± 787.5
Cooking (frying), min per month, mean ± SD	914.3 ± 1425.8	945.6 ± 974.9	292.1 ± 863.5	261 ± 765.4
Incense burning at home, n (%)				
Never	124 (55.1)	35 (34.3)	50 (45.9)	49 (45.8)
< Once a week	4 (1.8)	1 (1)	2 (1.8)	3 (2.8)
Once to trice a week	16 (7.1)	16 (15.7)	12 (11)	16 (15)
> Trice a week	81 (36)	50 (49)	45 (41.3)	39 (36.4)
Using range hood when cooking (frying), n (%)	203 (90.2)	93 (91.2)	95 (87.2)	91 (85)
Asthma, n (%)	3 (1.3)	2 (2)	3 (2.8)	5 (4.7)
Regular treatment for asthma, n (%)	1 (0.4)	0 (0)	1 (0.9)	4 (3.7)
Chronic airway diseases, n (%)	3 (1.3)	2 (2)	5 (4.6)	4 (3.7)
Regular treatment for chronic airway diseases, n (%)	1 (0.4)	0 (0)	4 (3.7)	2 (1.9)
FVC, mean ± SD, L	2.1 ± 0.4	2 ± 0.4	3 ± 0.6	2.7 ± 0.5
FEV1, mean ± SD, L	1.7 ± 0.3	1.6 ± 0.3	2.4 ± 0.5	2.1 ± 0.4
FEV1/FVC, mean ± SD, %	80.2 ± 5.8	79.9 ± 7.4	79.2 ± 7.9	77.5 ± 7
Obstructive ventilation*, n (%)	8 (3.6)	4 (3.9)	9 (8.3)	9 (8.4)
Total lung capacity (TLC), mean ± SD, L	4 ± 0.9	3.8 ± 0.9	5.1 ± 1	4.9 ± 0.9
Residual volume (RV), mean ± SD, L	1.9 ± 0.8	1.9 ± 1	2.1 ± 0.6	2.2 ± 0.9
RV/TLC, mean ± SD, %	47.3 ± 9.1	48.6 ± 9.9	41.4 ± 6.5	44.7 ± 9.2
DLco, mean ± SD, mL/mmHg/min	15.8 ± 3.2	15.9 ± 4	20.2 ± 4.7	18.7 ± 4.7
Alveolar volume (VA), mean ± SD, L	3.3 ± 0.6	3.4 ± 1	4.6 ± 0.9	4.3 ± 0.8
DLco/VA, mean ± SD, ml/min/mmHg/L	4.3 ± 0.7	4.4 ± 0.7	4.2 ± 0.8	4 ± 0.8
VA/TLC, mean ± SD, %	96.6 ± 0.9	96.7 ± 0.7	96.9 ± 0.7	97 ± 0.6
Number of visits				
1	80	41	30	42
2	67	27	40	30
3	78	34	39	35
Follow-up duration*, year	1.8 ± 0.6	1.8 ± 0.6	1.9 ± 0.6	1.8 ± 0.6

*SHS* second-hand smoke, *SD* standard deviation

*Pack-years was calculated only for smoker and ex-smoker

*The definition of obstructive ventilation was FEV1/FVC less than the value of lower limit of normal (LLN)

*Follow-up duration was calculated for the 350 participants having repeat visits

The distributions of exposures to PM_2.5_ and NO_2_ among the participants (Table 3) showed that the interquartile range of PM_2.5_ and NO_2_ levels were 8.1 μg/m^3^ and 5.4 ppb for long-term (lag one year) exposures and 10.2 μg/m^3^ and 8.2 ppb for short-term (lag one month) exposures. This pointed to a considerable variation in air pollution exposures across the residential areas of the participants. The correlation coefficient between PM_2.5_ and NO_2_ was 0.45 for one-year exposure and 0.66 for one-month exposure.

**Table 3:** Air pollution exposures before measurements of lung volume and diffusion capacity for the visits (n = 1079) of the elderly participants (n = 543) in this study, 2016 ~ 2018

	Mean	SD	Median	Q1	Q3	Interquartile range
One year before test						
PM_2.5_, μg/m^3^	24.35	5.17	23.93	21.05	29.18	8.13
NO_2_, ppb	21.81	6.63	21.30	19.26	24.63	5.37
One month before test						
PM_2.5_, μg/m^3^	21.80	7.43	20.56	16.36	26.55	10.19
NO_2_, ppb	20.75	7.21	20.09	16.00	24.23	8.24

*SD* standard deviation, *Q1* first quartile, *Q3* third quartile

The association between long-term exposure to air pollution and lung function indices (Tables 4 and 5) revealed that every IQR increase in exposure to NO_2_ was associated with an additional rate of decline in TLC, RV, RV/TLC, DLco, and VA/TLC of 1.82% (95% CI: − 2.76 to − 0.87%), 3.34% (95% CI: − 5.03 to − 1.62%), 1.58% (95%CI: − 2.62 to − 0.53%), 1.13% (95% CI: − 2.02 to − 0.23%), and 0.08% (95% CI: − 0.12 to − 0.04%) per year, respectively, indicating a detrimental effect of NO_2_ exposure on total lung volume and diffusion capacity. The effect of NO_2_ remained consistent after adjusting for PM_2.5_ exposure. On the other hand, during the follow-up period, every IQR increase in PM_2.5_ exposure was associated with an additional rise in DLco/VA of 1.53% (95% CI: 0.06–3.03%) per year. In the data analysis of spirometry, the PM_2.5_ and NO_2_ mutually adjusted model showed that each IQR increase in PM_2.5_ exposure was associated with an additional rate of reduction in the FEV1/FVC ratio by 0.82% (95% CI: − 1.38 to − 0.26%), indicating an obstructive ventilatory effect (Table 6). Although NO_2_ was significantly associated with FEV1, FVC, and FEV1/FVC ratio in single-pollutant model, the effect became non-significant after mutually adjusted for PM_2.5_, indicating the collinearity between air pollutants rather than the true independent effect of NO_2_ (Table 6). In summary, long-term NO_2_ exposure was associated with additional decline rates in TLC, RV, and RV/TLC, but not FEV1/FVC, suggesting a restrictive ventilation effect.

**Table 4:** Longitudinal association* between air pollution exposures and parameters of lung volume for visits (n = 792) done by the participating elderly (n = 420) in this study

	TLC			RV			RV/TLC		
	β (%)	95% CI	P-value	β (%)	95% CI	P-value	β (%)	95% CI	P-value
Model 1: PM_2.5_									
Cross-sectional	0.01	− 5.05 to 5.35	0.996	− 1.82	− 9.42 to 6.41	0.654	− 0.29	− 5.12 to 4.79	0.910
On rate of yearly change	− **2.15**	− **3.67 to **− **0.6**	**0.007**	− **3.68**	− **6.4 to **− **0.87**	**0.011**	− 1.54	− 3.22 to 0.17	0.078
Model 2: NO_2_									
Cross-sectional	− 0.09	− 3.86 to 3.84	0.965	− 2.17	− 8.45 to 4.53	0.515	− 1.46	− 5.34 to 2.58	0.474
On rate of yearly change	− **1.82**	− **2.76 to **− **0.87**	**0.0002**	− **3.34**	− **5.03 to **− **1.62**	**0.0002**	− **1.58**	− **2.62 to **− **0.53**	**0.003**
Model 3: PM_2.5_ + NO_2_									
PM_2.5_									
Cross-sectional	− 2.71	− 8.65 to 3.62	0.391	− 6.5	− 15.3 to 3.2	0.181	− 2.91	− 8.62 to 3.16	0.339
On rate of yearly change	− 0.46	− 2.47 to 1.59	0.655	− 0.38	− 4.02 to 3.4	0.842	0.14	− 2.08 to 2.41	0.901
NO_2_									
Cross-sectional	1.92	− 4.08 to 8.3	0.538	4.33	− 6.13 to 15.96	0.431	2.42	− 3.93 to 9.19	0.463
On rate of yearly change	− **1.69**	− **2.94 to **− **0.43**	**0.009**	− **3.32**	− **5.53 to **− **1.05**	**0.004**	− **1.69**	− **3.05 to -0.3**	**0.017**

Statistically significant (p-value < 0.05), estimates are shown in bold

*TLC* total lung capacity, *RV* residual volume

Adjusted for sex, age at baseline, time from baseline, body height, body weight, current smoke, ex-smoke, pack-years, second-hand smoke, cooking, use of range hood, incense, education, physician diagnosis or treatment for asthma or chronic airway diseases, dusty job history, obstructive ventilation, short-term (lag 1 month) exposure to air pollution, and hospitals

Coefficients were estimated for an interquartile range increase in the exposures to air pollutants (8.13 μg/m^3^ for PM_2.5_; 5.37 ppb for NO_2_)

**Table 5:** Longitudinal association* between air pollution exposures and parameters of diffusion capacity for visits (n = 1079) done by the participating elderly (n = 543) in this study

	Dlco			Dlco/VA			VA/TLC*		
	β (%)	95% CI	P-value	β (%)	95% CI	P-value	β (%)	95% CI	P-value
Model 1: PM_2.5_									
Cross-sectional	2.31	− 3.39 to 8.35	0.432	0.46	− 4.58 to 5.76	0.859	− 0.04	− 0.23 to 0.15	0.676
On rate of yearly change	− 0.61	− 2.19 to 0.99	0.454	**1.53**	**0.06 to 3.03**	**0.042**	− **0.09**	− **0.16 to **− **0.03**	**0.006**
Model 2: NO_2_									
Cross-sectional	3.38	− 0.3 to 7.19	0.072	1.5	− 1.67 to 4.78	0.357	− 0.04	− 0.18 to 0.11	0.645
On rate of yearly change	− **1.13**	− **2.02 to **− **0.23**	**0.014**	0.33	− 0.5 to 1.16	0.439	− **0.08**	− **0.12 to **− **0.04**	**0.0002**
Model 3: PM_2.5_ + NO_2_									
PM_2.5_									
Cross-sectional	− 0.57	− 7.12 to 6.43	0.868	1.77	− 4.27 to 8.19	0.569	− 0.11	− 0.34 to 0.13	0.362
On rate of yearly change	0.34	− 1.45 to 2.17	0.710	**1.63**	**0 to 3.28**	**0.051**	− 0.03	− 0.11 to 0.06	0.531
NO_2_									
Cross-sectional	1.58	− 4.36 to 7.9	0.609	− 2.34	− 7.48 to 3.08	0.389	0.02	− 0.22 to 0.26	0.863
On rate of yearly change	− **1.28**	− **2.3 to **− **0.24**	**0.016**	− 0.07	− 1 to 0.86	0.880	− **0.07**	− **0.12 to **− **0.02**	**0.009**

Statistically significant (p-value < 0.05), estimates are shown in bold

*Dlco* diffusing capacity for carbon monoxide, *VA* alveolar volume, *TLC* total lung capacity

Adjusted for sex, age at baseline, time from baseline, body height, body weight, current smoke, ex-smoke, pack-years, second-hand smoke, cooking, use of range hood, incense, education, physician diagnosis or treatment for asthma or chronic airway diseases, dusty job history, obstructive ventilation, short-term (lag 1 month) exposure to air pollution, and hospitals

Coefficients were estimated for an interquartile range increase in the exposures to air pollutants (8.13 μg/m^3^ for PM_2.5_; 5.37 ppb for NO_2_)

VA/TLC was calculated for 420 participants with TLC results provided by hospitals

**Table 6:** Longitudinal association* between air pollution exposures and parameters of forced lung function (n = 543)

	FVC			FEV1			FEV1/FVC		
	β (%)	95% CI	P-value	β (%)	95% CI	P-value	β (%)	95% CI	P-value
Model 1: PM_2.5_									
Cross-sectional	0.7	− 2.56 to 4.06	0.647	− 0.06	− 3.61 to 3.62	0.971	− 0.85	− 2.9 to 1.26	0.418
On rate of yearly change	0.18	− 0.78 to 1.16	0.712	− 0.43	− 1.32 to 0.47	0.347	− **0.82**	− **1.38 to **− **0.26**	**0.004**
Model 2: NO_2_									
Cross-sectional	**3.73**	**1.35 to 6.17**	**0.002**	**2.5**	**0.28 to 4.77**	**0.027**	− **1.68**	− **2.95 to **− **0.39**	**0.011**
On rate of yearly change	0.1	− 0.46 to 0.65	0.733	0.01	− 0.5 to 0.53	0.9627	− 0.1	− 0.42 to 0.22	0.5374
Model 3: PM_2.5_ + NO_2_									
PM_2.5_									
Cross-sectional	− 0.11	− 3.87 to 3.79	0.951	0.15	− 3.99 to 4.48	0.940	0.15	− 2.32 to 2.67	0.907
On rate of yearly change	0.27	-0.82 to 1.37	0.626	− 0.38	− 1.39 to 0.63	0.457	− **0.86**	− **1.49 to **− **0.23**	**0.008**
NO_2_									
Cross-sectional	0.68	− 3.13 to 4.64	0.730	− 0.68	− 4.19 to 2.95	0.708	− 1.47	− 3.57 to 0.68	0.179
On rate of yearly change	− 0.09	− 0.72 to 0.54	0.776	0.01	− 0.58 to 0.6	0.980	0.12	− 0.25 to 0.48	0.535

Statistically significant (p-value < 0.05), estimates are shown in bold

*FVC* forced vital capacity, *FEV1* forced expiratory volume in one second

Adjusted for sex, age at baseline, time from baseline, body height, body weight, current smoke, ex-smoke, pack-years, second-hand smoke, cooking, use of range hood, incense, education, physician diagnosis or treatment for asthma or chronic airway diseases, dusty job history, obstructive ventilation, short-term (lag 1 month) exposure to air pollution, and hospitals

Coefficients were estimated for an interquartile range increase in the exposures to air pollutants (8.13 μg/m^3^ for PM_2.5_; 5.37 ppb for NO_2_)

## Discussion

In this prospective cohort study on the elderly, exposure to ambient nitric dioxide accelerates the declines in total lung capacity, residual volume, ratio of residual volume to total lung capacity, diffusion capacity of the lungs, and the ratio of alveolar volume to total lung capacity. The findings suggest that air pollution exposure, as indicated by nitric dioxide, may cause a progression of restrictive ventilatory pattern accompanied by a decline in gas diffusion capacity.

A previous human experimental study showed that acute exposure to NO_2_ decreased DLco [30], while a long-term exposure study in dogs showed that NO_2_ decreased DLco but had no effect on TLC [19]. The experimental studies described above typically exposed high concentrations (~ 600–5000 ppb), much higher than the ambient concentrations in our study (~ 20 ppb on average), suggesting that the observed effects in our study may be through mechanisms other than NO_2_ itself. NO_2_ is usually considered a proxy of exposure to traffic-related air pollution [44]. A study on German children aged 5–7 years showed that a lower concentration of ambient total suspended particles was associated with better TLC. But the effect was diminished when children lived near high-traffic roads, indicating that traffic-related air pollution exposure has an additional impact on TLC [27]. Such findings are similar to ours. While both PM_2.5_ and NO_2_ significantly enhance TLC decline in single pollutant models, only NO_2_ remains statistically significant after the mutual adjustment of these two pollutants, indicating that traffic-related air pollution may be the critical source of exposure.

The observed NO_2_ effects on total lung capacity and diffusion capacity suggest the alveolar region as the site of its action. As a highly reactive gas, NO_2_ rapidly reacts with substrates (e.g., urate, ascorbate, and glutathione) in the epithelial lining fluid (ELF) of the airways, where inhaled NO_2_ is reduced to nitrite [45]. In the tracheobronchial region, the ELF is thick enough to prevent direct penetration of NO_2_. However, the thickness of the ELF drops sharply in the alveolar region, allowing NO_2_ diffusion into tissues. Although predicted models show a higher tissue dose of NO_2_ in the alveolar region compared to the tracheobronchial region, the health consequences are still unknown [45]. A murine study has demonstrated that long-term (18–27 months) exposures to NO_2_ cause morphologic changes in the centri-acinar region, including hypertrophy and proliferation of the terminal airway epithelium, thickening of the alveolar duct and basement membrane, and increased collagen fibers in the interstitium [46]. Interstitial fibrosis usually requires a longer time for recovery. Continuous exposure may cause an accumulation of lesions and lead to progressive functional impairment.

Clinically, the ratio of RV to TLC is an alternative indicator for obstructive lung diseases, such as asthma, chronic obstructive pulmonary disease (COPD), and emphysema [11]. Air trapping and decreased elastic recoil of the lungs increase the RV/TLC ratio [11], which also increases with age [47]. An elevated ratio is considered a risk factor for all-cause mortality in COPD patients [48]. This study shows a negative association between NO_2_ exposure and the annual change in TLC and RV/TLC, suggesting an additional restrictive process or an increase in elastic recoil in the lungs. The observed restrictive ventilatory effect of NO_2_ is similar to the result from the large Dutch population-based LifeLines Cohort Study [49]. They found that chronic exposure to NO_2_ had a greater effect on FVC decrease than FEV1. Moreover, NO_2_ exposure concentration was also positively correlated with the FEV1/FVC ratio, implying a restrictive ventilatory effect. However, whether the observed effects of NO_2_ on RV/TLC have clinical implications and health consequence is still unknown and requires further studies.

The NO_2_ causes an additional rate of decline in DLco, but not a decrease in DLco/VA, suggesting that the effect of NO_2_ on diffusion capacity is mainly due to decreased alveolar volume. Conceptually, the loss of DLco may be higher than that of VA in parenchymal lung abnormality, which may lead to a reduction in the DLco/VA ratio [11]. However, real-world data from patients with IPF shows that the mean DLco/VA is within the normal range regardless of the severity of restriction, whereas DLco decreases with increasing restriction [50]. Therefore, although DLco/VA aids in understanding the mechanism of DLco abnormality, current recommendations favor DLco over DLco/VA in interpreting lung function [11].

The observed association between PM_2.5_ exposure and an additional rate of decrease in FEV1/FVC is in line with previous studies on spirometry and the risk of chronic obstructive pulmonary diseases. Guo et al. [10] analyzed a large cohort of health exams in Taiwan and reported that every 5 μg/m^3^ increase in PM_2.5_ exposure was associated with an FEV1/FVC decline rate of 0.21% per year. In addition, they reported a higher risk for COPD development among participants exposed to the fourth (HR 1.39), third (HR 1.30), and second (HR1.23) quartiles of PM_2.5_, compared to those in the lowest quartile of PM_2.5_. Another cross-sectional study in China also showed that PM_2.5_ exposure was associated with an increased prevalence of COPD [51]. These findings may support the inference that PM_2.5_ contributes to obstructive ventilatory dysfunction.

In this study, PM_2.5_ is associated with an additional rate of increase in the carbon monoxide transfer coefficient (DLco/VA). The DLco/VA increases when VA decreases due to submaximal inflation, or when pulmonary blood flow increases [52]. Inspiratory muscle weakness may cause submaximal inflation and low VA [52]. Although there is still no direct evidence that PM_2.5_ exposure can cause inspiratory muscle weakness, some studies have shown that PM_2.5_ has a negative impact on skeletal muscle mass [53] and handgrip strength [54]. In addition, the strength of respiratory muscles, especially inspiratory muscles, is significantly related to skeletal muscle mass and handgrip strength [55]. It can therefore be posited that the observed effect of PM_2.5_ on DLco/VA may be mediated by a decrease in respiratory muscle strength, although this requires more evidence.

The VA/TLC is an indicator for ventilatory inhomogeneity, when VA is measured by the single breath tracer gas method and TLC is by body plethysmography [56]. Ventilatory inhomogeneity is commonly noted in people with emphysema or bullous lung disease. Emphysema can cause an abnormality in the alveolo-capillary membrane (low DLco and low DLco/VA) and an increase in ventilatory inhomogeneity (low VA/TLC). A combination of low DLco, normal DLco/VA, and low VA/TLC suggests an increase in inaccessible lung parts, such as in blebs and bullae. However, in this study, both VA and TLC are measured by the single breath inert trace gas dilution method, which does not allow for the assessment of blebs and bullae.

The difference between VA and TLC in this study refers to the anatomic dead-space or the volume of conducting airways, which is calculated by the Fowler method [57]. In short, the observed effects of NO_2_ exposure on the longitudinal reduction in VA/TLC suggest an increase in the proportion of anatomical death space to TLC. A previous study has shown that the ratio of anatomic dead-space to TLC in patients with IPF is higher than that in healthy controls, but it does not correlate with disease severity [58]. Although the effect size of the VA/TLC ratio is small (-0.08% for an IQR increase in NO_2_ exposure) and is unlikely to represent any meaningful clinical effect, the findings here suggest an effect of air pollution on restrictive lung disease.

This study has its strengths. First, the study cohort has an advantage in examining the impact of air pollution on lung function decline and restrictive lung disease because the participants come from five geographical areas with different levels of air pollution. Also, because their average age is very high, they are more vulnerable to air pollution and have a higher risk of pulmonary fibrosis. Second, in the statistical analysis, air pollution and co-pollutants have been mutually adjusted for the short-term, which allowed for extracting the observed long-term effects of nitric dioxide without being confounded by PM_2.5_. Third, measurements and follow-up of static lung volume and diffusion capacity have been performed in the same hospital-based lung function laboratory for all of the participants. This ensures the accuracy and reliability of the test.

On the other hand, some limitations are noteworthy. First, the single breath helium dilution method used in this study may underestimate total lung volume, particularly in patients with significant obstructive airway diseases. Severe airway obstruction is commonly associated with air trapping and bullous formation in the lungs, which cause an inhomogeneous distribution of tracing gas. Given that only 5% of the participants have an obstructive ventilatory defect and that adjustments have been made for this in the statistical models, bias related to the test method is small and less likely to change the conclusions.

Second, the DLco value is not adjusted to hemoglobin level. Reductions in hemoglobin and lung volume can both decrease DLco. A previous cross-sectional study shows the association of long-term (1-year) NO_2_ and anemia and the reduction of 0.81 g/dl in hemoglobulin for an IQR (9.6 ppb) increase in NO_2_. The current study does not determine whether or not the observed effect of NO_2_ on DLco is mediated by hemoglobin. However, if the observed DLco effect is related to a decline in hemoglobulin, there should also be a similar effect on the decline rate of DLco/VA because the alveolar volume is not affected by anemia. Furthermore, the non-significant positive association between NO_2_ exposure and DLco/VA suggests that hemoglobin does not play a major role in the observed decline rate in DLco.

Third, an unmeasured error in exposure assessment of air pollution is probable. Nonetheless, the accuracy and precision of the spatial–temporal modeling are high for NO_2_ and PM_2.5_ in previous validation studies [41, 42]. Unlike working and school-age people, who may be exposed to different levels of air pollution in the workplace or in school, estimation of outdoor pollution in a residential address is more relevant for the elderly. In addition, outdoor levels may not represent indoor levels. Several important indoor air pollution sources, including second-hand smoke, cooking fume, and incense, have been evaluated and adjusted for. Thus, the exposure error is probably small and unlikely to introduce non-differential misclassification or cause an underestimation of the observed effects.

## Conclusions

In conclusion, long-term exposure to ambient NO_2_ accelerates the decline in total lung volume and diffusion capacity of the lungs. Impaired diffusion capacity is related to the loss of lung volume. These findings suggest that air pollution may be a risk factor for restrictive lung disorders.

## Data Availability

The datasets used and/or analyzed during the current study are available from the corresponding author on reasonable request.

## Abbreviations

PM_2.5_: Particulate matter with an aerodynamic diameter ≤ 2.5 μm
NO_2_: Nitrogen dioxide
IQR: Inter-quartile range
FVC: Forced vital capacity
FEV1: Forced expiratory volume in one second
TLC: Total lung capacity
RV: Residual volume
DLco: Diffusion capacity of carbon monoxide
VA: Alveolar volume
